# T184. BRAIN-WIDE FUNCTIONAL DYSCONNECTIVITY IN SCHIZOPHRENIA: PARSING DIATHESIS, RESILIENCE AND THE EFFECTS OF CLINICAL EXPRESSION

**DOI:** 10.1093/schbul/sby016.460

**Published:** 2018-04-01

**Authors:** Shuixia Guo, Wei Zhao, Haojuan Tao, Liu Zhening, Lena Palaniyappan

**Affiliations:** 1 Hunan Normal University; 2 Mental Health Institute, The Second Xiangya Hospital of Central South University; 3 University of Western Ontario

## Abstract

**Background:**

The functional dysconnectivity observed from resting-state fMRI studies in schizophrenia is also seen in unaffected siblings indicating its association with the genetic diathesis of the illness. Nevertheless, when compared to patients, the extent of dysconnectivity appears to be limited both in spatial distribution and magnitude in siblings, suggesting that some of the abnormalities could be exclusively linked to the clinical expression or treatment effect rather than genetic diathesis. We investigated brain-wide functional connectivity using a graph theory approach to apportion resting-state dysconnectivity into components that represent genetic diathesis, clinical expression or treatment effect and resilience.

**Methods:**

Resting state functional MRI data acquired from 116 subjects (28 patients with schizophrenia, 28 unaffected siblings and 60 matched healthy controls). Based on Dosenbach’s atlas applied to 6 minutes (180 time points with TR=2 s) of eyes-open resting fMRI scan, we extracted time series of 160 functional network nodes. After constructing a 160*160 functional network, we investigated between-group differences in strength and diversity of functional connectivity and topological properties of undirected graphs constructed from thresholded correlation matrices. We also used Support Vector Machine approach to estimate the ability of functional connectivity metrics to discriminate the three groups from each other.

**Results:**

Using ANOVA [FDR corrected p<0.05], we found 88 out of 12720 pairs of functional links to be significantly different among the three groups. 48.8% of these 88 links included nodes from the Default Mode Network (DMN), with the largest portion of these involving Salience Network/DMN connectivity (14.8%). Post-hoc t tests revealed that 62.5% of these disconnected links were associated with genetic diathesis of schizophrenia (i.e. both patients and siblings showing same direction of significant post-hoc difference compared to HC) and 21.6% were associated with clinical expression or treatment effect (i.e. patients differed from siblings and healthy controls, but no difference between controls and siblings). Topologically, we observed increased degree, clustering coefficient and global efficiency but reduced local efficiency in the sibling group compared to both patients and controls, indicating a resilience (or compensation) effect. Support vector machine analysis revealed a high degree of accuracy when classifying the genetically predisposed (patients and siblings) vs. healthy controls (Area Under the Curve - AUC 0.97) or the patient groups vs. healthy controls (AUC 0.97) but not when discriminating patients vs. siblings (AUC 0.58)

**Discussion:**

A large portion of the resting-state functional dysconnectivity seen in patients with schizophrenia represent a genetic diathesis effect. The most prominent network level disruption in this context is the dysconnectivity among nodes of the default-mode and salience networks. Despite their predisposition, unaffected siblings show a pattern of resilience in the emergent connectomic topology. Our findings could potentially help refine imaging genetics approaches currently used in the pursuit of the pathophysiology of schizophrenia.

